# Resection of small plexiform neurofibromas in neurofibromatosis type 1 children

**DOI:** 10.1186/1477-7819-3-6

**Published:** 2005-01-31

**Authors:** Reinhard E Friedrich, Rainer Schmelzle, Melanie Hartmann, Carsten Fünsterer, Victor-F Mautner

**Affiliations:** 1 Department of Maxillofacial Surgery, Universitätskrankenhaus Hamburg-Eppendorf, Martinistr. 52, 20246 Hamburg, Germany; 2 MRI Institute, Othmarscher Kirchenweg 166, 22763 Hamburg, Germany

## Abstract

**Background:**

Plexiform neurofibromas (PNF) are benign tumors of the peripheral nerve which mostly develop in patients with neurofibromatosis type 1 (NF1). Surgical interventions are usually not applied to children with small tumors. These are rather restricted to debulking of larger tumors in adults that cause clinical complications or aesthetic disfigurement. In most cases, a total resection of PNF is not possible due to the network-like growth of the tumors.

**Patients and methods:**

Early surgical intervention was carried out for 9 small PNFs in 7 NF1 children. Tumor resection was performed following the graphical delineation of the affected skin and according the MRI findings.

**Results:**

Total resection was achieved for all 9 PNF without causing any neurological or organic deficit. Annual magnetic resonance tomography over a period of four years did not reveal any relapse of the tumors.

**Conclusions:**

Early surgical intervention for small superficial PNFs in NF1 children have various advantages and may especially be considered a strategy to prevent progression.

## Background

Plexiform neurofibromas (PNF) are benign tumors originating from nerve sheath cells, subcutaneous, or visceral peripheral nerves and can involve multiple fascicles [1]. PNF occur almost exclusively in patients with NF1, an autosomal dominant disorder caused by defect of one allele of the tumor suppressor gene, *NF1 *on 17q [2-5]. At least 30% of NF1 patients suffer from PNF [2,6,7], which are often present at birth and progress during the first years of life. The growth rate and pattern of PNF vary to a large extent and their growth spurts are unpredictable. PNF can arise in various parts of the body, for example as anterior mediastinal masses, sciatic nerve lesions with pelvic extension, or perirectal plexiform and uterine tumors, often leading to severe clinical complications [7]. Especially tumors occurring in the head and neck area often lead to facial disfigurement and functional deficits. Due to large size of the tumors and involvement of multiple fascicles of nerves and tissues, the risk of neurological and functional destruction upon tumor resection is high. Surgical interventions are thus commonly postponed as long as possible. In addition, most surgical interventions are limited to debulking and rests of tumors often re-grow afterwards leading to the requirement of repeated intervention [8-10].

Previously we reported that PNF can be distinguished into three growth categories using magnetic resonance imaging (MRI): superficial, displacing and invasive. Superficial PNF arise from subcutaneous or cutaneous nerves and may remain within the upper layer of the skin – usually not involving major nerves [10]. Subtotal and total resection without functional destruction is often possible for superficial PNF, as demonstrated in our recent study [11]. In contrast, invasive PNF infiltrate multiple tissue planes and are thus much more difficult or impossible to resect. Superficial PNF show progressive growth and it is unknown whether or not a superficial PNF may change to displacing or invasive types. Early resection of small superficial PNF may thus be considered as an advantageous treatment option.

In this study, we present the results of early intervention for 9 PNFs in 7 NF1 children aged 3 to 15 years.

## Patients and methods

The 7 children were examined in our NF-Clinic in the Department of Maxillofacial Surgery, University Hospital Eppendorf, Hamburg. Diagnosis of NF1 was based on the NIH criteria [6]. Informed consent was obtained from parents of these children. All patients received dermatological, neurological and ophthalmological examinations as well as an ultrasound of abdominal organs. PNF were diagnosed based on the following indications: subcutaneous location on palpation, associated with thickening of the skin, local hypertrophy, hair excess and hyperpigmentation on inspection, and prepubertal occurrence from the medical report. Magnetic resonance image (MRI) was done for the tumor regions at 1.5 Tesla with T1- and T2- weighted sequences including a short-tau inversion-recovery (STIR) sequence. Ultra rapid half Fourier single-shot turbo spin-echo (HASTE) sequences were used for imaging the trunk. Intravenous contrast medium was given to all patients. Tumor resection was performed following the graphical delineation of the affected skin and according the MRI findings. Tumor resection included the epidermal and subcutaneous layer with safety margins of 1 cm in all directions. Exploration of the underlying muscles was mandatory.

## Results

**Figure 1:**
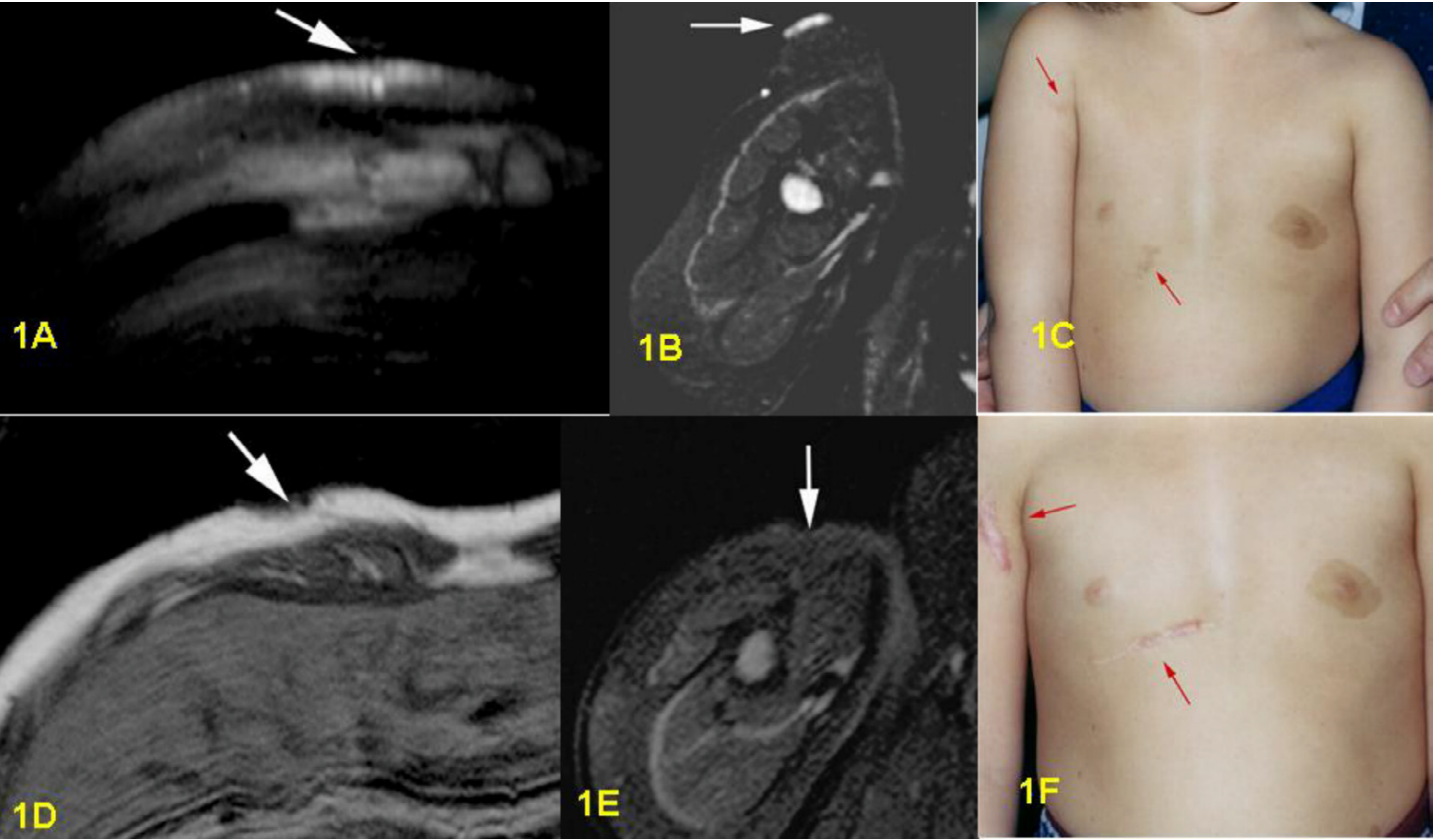
1A: Pre-surgical T2-weighted STIR-sequence, axial section: in right ventral chest wall bright superficial thickening of cutis and subcutis without involvement of muscles. 1B: Pre-surgical T2-weighted STIR-sequence, axial section: very bright, flat superficial cutaneous PNF in proximal part of right upper arm. 1C: Pre-surgical clinical frontal view of both PNF on right upper arm and thorax wall. 1D: Post-surgical T1-weighted sequence, axial section: small defect in cutis and subcutis, no PNF visible anymore. 1E: Post-surgical T2-weighted STIR-sequence, axial section: complete removal of tumor with hypointensive induration. 1F: Post-surgical clinical frontal view of both scars on right upper arm and thorax wall.

**Figure 2:**
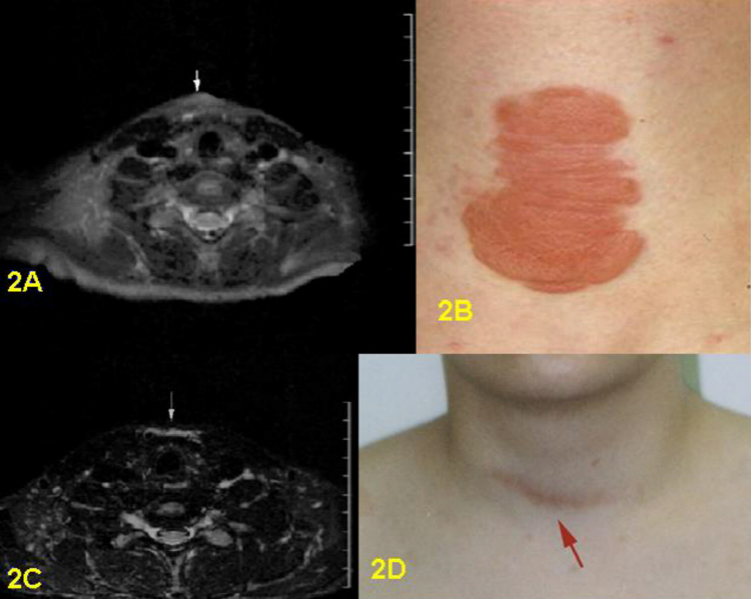
2A: Pre-surgical T2-weighted STIR-sequence, axial section: superficial tumor covering the laryngeal prominence. 2B: Pre-surgical clinical frontal view of PNF of ventral neck. 2C: Post-surgical T2-weighted STIR-sequence, axial section: scar tissue praelaryngeal visible. 2D: Post-surgical clinical frontal view of laryngeal scar.

**Figure 3:**
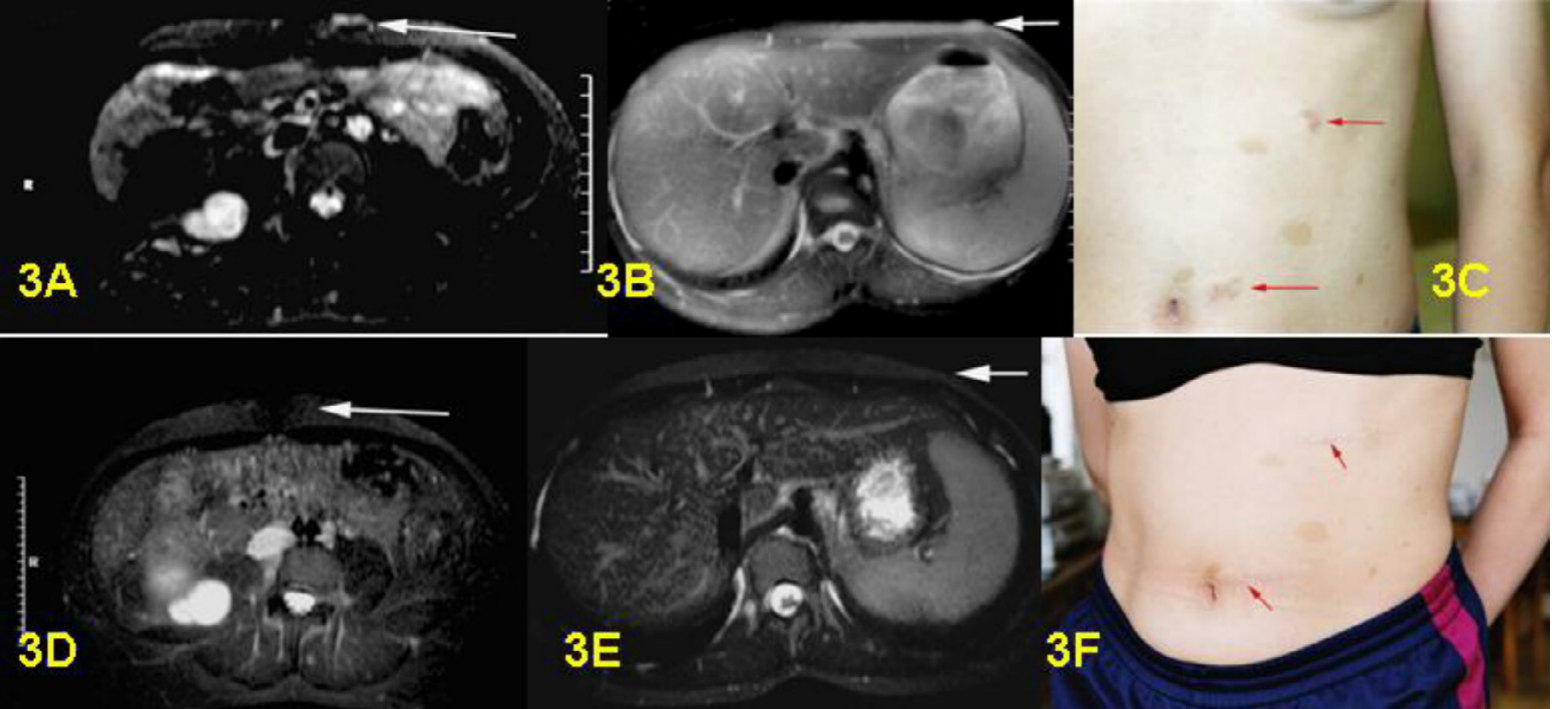
3A: Pre-surgical T2-weighted STIR sequence of left ventral abdominal wall, paraumbilical. Bright plexiform neurofibroma with thickening of cutis without involvement of muscle. 3B: Pre-surgical T2-weighted STIR sequence, axial section of left ventral abdominal wall, costal margin. Small bright plexiform neurofibroma in cutis and subcutis ventral left. 3C: Pre-surgical clinical frontal view of abdomen with both superficial plexiform neurofibromas visible. 3D: Post-surgical MRI control from A. Complete removal of plexiform neurofibroma. 3E: Post-surgical MRI control from B. Complete removal of plexiform neurofibroma. 3F: Post-surgical clinical frontal view of abdomen. Only two visible scars left after surgery.

**Figure 4:**
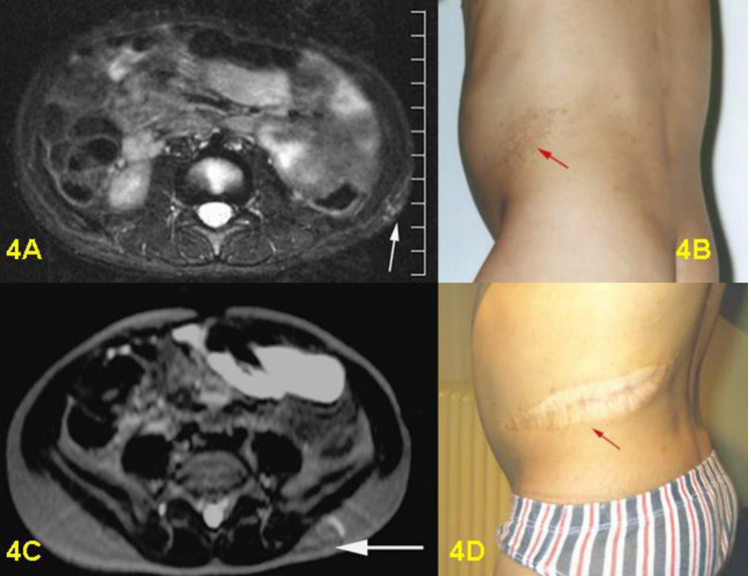
4A: Pre-surgical T2-weighted STIR-sequence, axial section: discrete signs of flat funicular, cutaneous and subcutaneous PNF near the left iliac crest with involvement of soft tissue, but without visible infiltration of abdominal muscles. 4B: Pre-surgical clinical view of PNF in the left flank. 4C: Post-surgical T2-weighted Haste-sequence: complete resection of tumor, only a smooth fibrous post-surgical induration and small scar can be identified. 4D: Post-surgical clinical view of hypertrophic scar.

**Figure 5:**
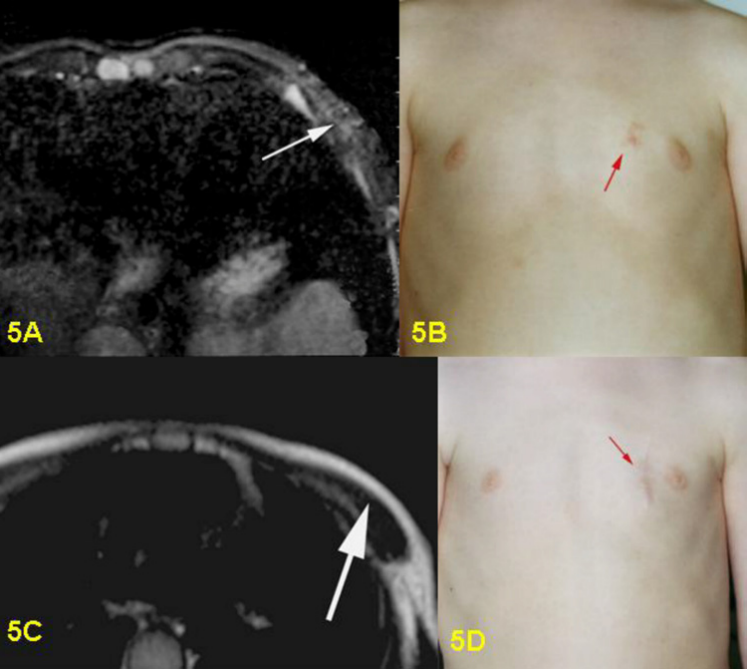
5A: Pre-surgical T2-weighted STIR-sequence, axial section; small nodular hyperintensive PNF in the cutis and subcutis on left ventral chest wall. 5B: Pre-surgical clinical view of PNF in left chest wall, parasternal. 5C: Post-surgical T2-weighted Turbo Spin Echo-sequence, axial section: tumor no longer visible, only adipose tissue visible in the subcutis. 5D: Post-surgical clinical view of scar.

**Figure 6:**
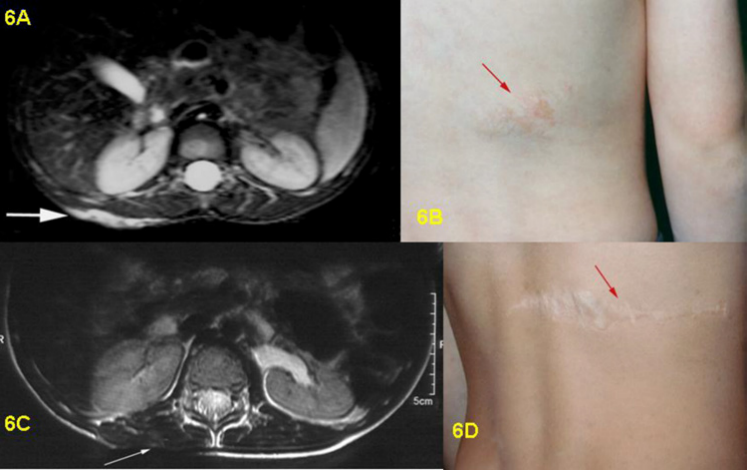
6A: Pre-surgical T2-weighted STIR-sequence, transversal section: bright, flat cutaneous and subcutaneous PNF of right back without involvement of abdominal wall and muscles. 6B: Pre-surgical clinical view of PNF on right back with hypertrichosis. 6C: Post-surgical T2-weighted Turbo Spin Echo-sequence, axial section: complete removal of PNF, thin scar, no subcutaneous fatty tissue visible in scan. 6D: Post-surgical clinical view of scar after tumor removal on back.

**Figure 7:**
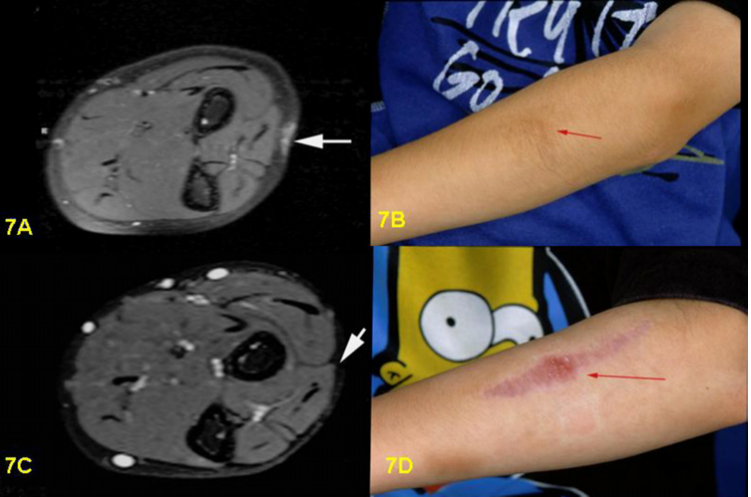
7A: Pre-surgical T2-weighted STIR-sequence, transversal section: Cutaneous and subcutaneous PNF of forearm, bright signal without involvement of muscles or fascia. 7B: Pre-surigcal clinical view of hyperpigmented PNF of left forearm. 7C: Post-surgical T2-weighted STIR-sequence, axial section: complete removal of tumor. 7D: Post-surgical clinical view of scar on left forearm.

**Figure 8:**
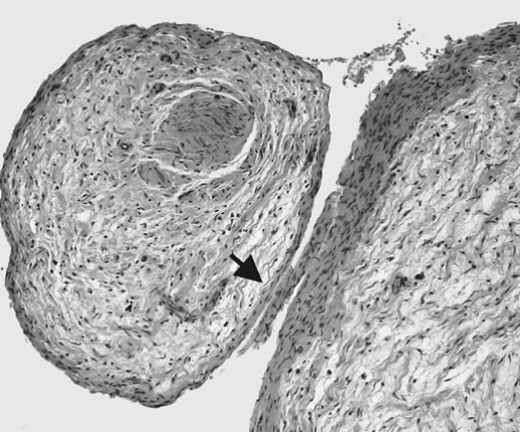
**Photomicrograph of PNF. **Plexiform neurofibroma WHO grade I invading fascicles of a peripheral nerve. Tumor growth is primarily confined to the endoneurial space; the perineurium (arrow) forms a natural border for the tumor. Note the loose myoxid texture of the tumor tissue

Seven NF1 children, five boys and two girls, aged 3 to 15 years were included in this study. All of them met the NIH diagnostic criteria for NF1 [6]. PNF was initially diagnosed clinically and further ascertained by MRI (Figs. 1, 2, 3, 4, 5, 6, 7). Five tumors were histologically confirmed as PNF (Fig. 8). Other four were reported as diffuse neurofibromas due to lack of nerve fascicle in the examined sections. However, these tumors were parts of PNF. Five PNF in four children exhibited hyperpigmentation while the other two had hair excess in the tumor area. Two children had two and the other five had one PNF each. Size of tumors varied from 2 cm to 8 cm in greatest diameter (table 1). All PNF were superficial based on MRI. The tumor location was variable (table 1). Neither functional deficit nor pain was caused by the tumors at the time of surgery. Neither was there significant aesthetic disfigurement caused by the tumors.

**Table 1:** Clinical information

**Patient number**	**Sex**	**Age at surgery**	**Tumor location**	**Hyper pigmentation**	**hair growth**	**Size (greatest diameter)**	**Histology**	**post-operative MRI**	**Annual MRI follow up**
1	f	5	upper arm right	no	no	4 cm	PNF	yes	yes
		5	thorax right	yes	no	4 cm	PNF	yes	yes
2	m	15	Adams apple	no	no	5 cm	PNF	no	yes
3	f	14	rib bow left	no	no	4 cm	PNF	no	yes
		14	paraumbilical left	no	no	3 cm	PNF	no	yes
4	m	3	thigh left	yes	yes	7 cm	PNF	no	yes
5	m	4	thorax left	yes	no	2 cm	PNF	yes	yes
6	m	7	back right	yes	yes	8 cm	PNF	yes	yes
7	m	11	lower arm left	yes	no	5 cm	PNF	no	only 2003

In 2001, all seven children underwent surgical intervention for their nine PNF. During the mobilization of the underlying skin layers, three patients showed enlarged nerves running from subcutis to superficial muscle layers, which were identified and additionally resected. These enlarged nerves were proven to be PNF in all cases. Primary wound closure was achieved following the mobilization of the marginal skin. Healing followed without complications in all cases. The scars are relatively small and no hypertrophy was observed. The intervention was well tolerated by all children and recovery occurred within a few days.

Postoperative MRI was done for three children. Six children underwent follow-up examinations annually by MRI for three years. One child was only examined in 2003, two years after the operation. MRI revealed successful total resection for all 9 tumors (Figs. 1, 2, 3, 4, 5, 6, 7). So far, no tumor re-growth has been detected in any of the children.

## Discussion

In this study we demonstrated that small superficial PNF can be completely resected, leaving scars without hypertrophy. Operation is relatively uncomplicated, allows healing by primary intention, requires only a few days of hospitalization and is thus no burden for even the youngsters. Within the clinical and radiological follow-up period of three years there was no re-growth of the tumors. However, it cannot be excluded that all tumor cells have been resected. Therefore those patients warrant further annual clinical examination.

The scars in some children may seem more obvious and disfiguring after the operation. However, in comparison to the complications the tumors may cause later, they are only of minor concern. Furthermore, these scars may be corrected later surgically.

Without resection, these small PNF are likely to grow continuously to a large or even very large size which often causes aesthetic disfigurement, functional deficits and pain. Resection of large tumors is much more difficult and total resection is usually not possible any more. Currently it is not clarified whether or not growth patterns of PNF might possibly change from superficial to displacing or to invasive types over time. Early surgical intervention of small superficial PNF may thus be considered as a preventive strategy for later disfigurement and functional deficits.

Adequate diagnosis of NF1 and thus small PNF in the pediatric age group is challenging. Children usually do not complain about subtle pain or discomfort in the affected skin region. Even in the presence of some symptoms parents are often not aware of the origin of the tumor. Only small PNF of the face and neck causing disfigurement in children tend to be recognized early. On the other hand, small PNF of the trunk and extremities which present with subtle hyperpigmentation, hair excess and palpable tumor tissue are frequently not noticed by the physicians. These tumors are rarely diagnosed correctly as PNF. In our cohort only one child received the diagnosis of a PNF correctly by the referring family physician. For better management and adequate treatment, efforts should be made to diagnose NF1 and PNF in their early stage.

## Conclusions

Early surgical intervention of small superficial PNFs is uncomplicated, without burden for even the youngsters and enables total resection of the tumors. It may be considered as a preventive strategy for later disfigurement and functional deficits.

## Competing interests

The authors declare that they have no competing interests.

## Authors' contributions

**REF **and **RS **carried out the surgical intervention for the tumors and post-operational care of the patients.

**CF **carried out the MRI and was involved in the diagnosis of PNF.

**MH **and **VFM **did the diagnosis and were responsible for management and consulting of the patients.

**REF **and **VFM **designed the study and prepared the manuscript.

All authors read and approved the final version of the manuscript.
